# Supplementary material for: The role of geography and host abundance in the distribution of parasitoids of an alien pest
Source: PeerJ. 2016 Jan 18;4:e1592. doi: 10.7717/peerj.1592 (PMC4727958; doi:10.7717/peerj.1592)
Supplement: Data S1 [file peerj-04-1592-s001.docx]

Raw data:

| ID | Species | Individuals | Host | Altitude | Latitude |
| --- | --- | --- | --- | --- | --- |
| 1 | 3 | 18 | 244 | 442 | 50.1222 |
| 2 | 2 | 42 | 242 | 447 | 50.236 |
| 3 | 1 | 8 | 165 | 473 | 49.4861 |
| 4 | 3 | 31 | 307 | 323 | 49.7476 |
| 5 | 2 | 27 | 408 | 185 | 50.1596 |
| 6 | 2 | 4 | 322 | 195 | 50.5862 |
| 7 | 1 | 8 | 255 | 327 | 49.8238 |
| 8 | 1 | 5 | 171 | 223 | 50.2866 |
| 9 | 2 | 50 | 876 | 195 | 50.0285 |
| 10 | 4 | 68 | 1091 | 208 | 49.9684 |
| 11 | 2 | 25 | 393 | 193 | 50.1867 |
| 12 | 1 | 11 | 229 | 561 | 49.5554 |
| 13 | 3 | 14 | 147 | 396 | 50.1887 |
| 14 | 1 | 5 | 287 | 382 | 50.619 |
| 15 | 1 | 1 | 54 | 360 | 50.7916 |
| 16 | 2 | 5 | 90 | 451 | 50.6092 |
| 17 | 1 | 3 | 48 | 235 | 50.2099 |
| 18 | 1 | 17 | 153 | 414 | 50.4128 |
| 19 | 4 | 21 | 358 | 221 | 50.0385 |
| 20 | 1 | 81 | 458 | 575 | 49.7097 |
| 21 | 3 | 27 | 250 | 454 | 49.666 |
| 22 | 2 | 5 | 597 | 330 | 49.8711 |
| 23 | 2 | 9 | 518 | 347 | 49.8739 |
| 24 | 1 | 3 | 264 | 495 | 50.0967 |
| 25 | 1 | 13 | 294 | 438 | 49.3286 |
| 26 | 3 | 9 | 214 | 203 | 49.195 |
| 27 | 1 | 35 | 1101 | 207 | 49.1149 |
| 28 | 4 | 118 | 1205 | 204 | 49.1277 |
| 29 | 3 | 43 | 204 | 173 | 48.7978 |
| 30 | 2 | 35 | 240 | 184 | 48.9552 |
| 31 | 2 | 19 | 272 | 290 | 48.856 |
| 32 | 2 | 4 | 211 | 178 | 48.9872 |
| 33 | 1 | 3 | 218 | 230 | 49.2298 |
| 34 | 1 | 8 | 243 | 372 | 49.5903 |
| 35 | 2 | 36 | 281 | 258 | 49.9172 |
